# Mesenchymal Stem Cell–Immune Cell Interaction and Related Modulations for Bone Tissue Engineering

**DOI:** 10.1155/2022/7153584

**Published:** 2022-02-01

**Authors:** Renxin Chen, Zhuowen Hao, Yi Wang, Hongzhen Zhu, Yingkun Hu, Tianhong Chen, Peng Zhang, Jingfeng Li

**Affiliations:** ^1^Department of Orthopedics, Zhongnan Hospital of Wuhan University, Wuhan 430071, China; ^2^Department of Orthopedics, Suzhou Science and Technology Town Hospital, The Affiliated Suzhou Science and Technology Town Hospital of Nanjing Medical University, Suzhou 215153, China

## Abstract

Critical bone defects and related delayed union and nonunion are still worldwide problems to be solved. Bone tissue engineering is mainly aimed at achieving satisfactory bone reconstruction. Mesenchymal stem cells (MSCs) are a kind of pluripotent stem cells that can differentiate into bone cells and can be used as one of the key pillars of bone tissue engineering. In recent decades, immune responses play an important role in bone regeneration. Innate immune responses provide a suitable inflammatory microenvironment for bone regeneration and initiate bone regeneration in the early stage of fracture repair. Adaptive immune responses maintain bone regeneration and bone remodeling. MSCs and immune cells regulate each other. All kinds of immune cells and secreted cytokines can regulate the migration, proliferation, and osteogenic differentiation of MSCs, which have a strong immunomodulatory ability to these immune cells. This review mainly introduces the interaction between MSCs and immune cells on bone regeneration and its potential mechanism, and discusses the practical application in bone tissue engineering by modulating this kind of cell-to-cell crosstalk. Thus, an in-depth understanding of these principles of bone immunology can provide a new way for bone tissue engineering.

## 1. Introduction

Although bone tissue can undergo self-healing during bone repair, critical bone defects caused by severe fracture, tumor excision, congenital defects, arthritis, and osteoporosis remain a global concern because regenerative requirement exceeds the bone's capacity to heal itself [1, 2]. Autologous bone grafting is the gold standard for surgical bone repair, but it is limited because of potential complications, including chronic pain, infections, and hematomas [3, 4]. Allogenic bone grafting and xenogenic bone grafting are alternative approaches for critical bone defects, but they show predominant disadvantages, including high costs and risks of disease transmission and immune rejection [5, 6]. However, these bone grafts have limitations, so novel bone regeneration strategies for critical bone defects should be developed.

Bone tissue engineering is a potential strategy for critical bone defects, which are composed of four pillars, namely, biomaterial scaffolds, stem cells, bioactive factors, and biophysical stimuli [7]. However, most current studies have mainly focused on osteogenesis and angiogenesis during bone healing because osteogenesis stimulates the deposition of collagen and hydroxyapatite; angiogenesis promotes the delivery of oxygen and nutrients for bone cells to exert their functions [8, 9]. However, the immune system elicits indispensable effects on bone regeneration through which immune cells and secreted cytokines are essential [10].

During bone repair, acute inflammation caused by damage factors such as trauma is generally the first inflammation stage, which is followed by a degenerative or regenerative stage determined by the crosstalk between immune cells and bone cells [11]. In bone tissue engineering, stem cells are one of the pivotal pillars for bone tissue engineering; among them, mesenchymal stem cells (MSCs) have been widely used for bone regeneration [12]. MSCs can be regulated by various immune cells to migrate and differentiate, and they show immunomodulatory functions to interact with various immune cells (such as T cells and macrophages) by cell-cell contact or secreted factors [13, 14].

In this review, the osteoinductive effects of immune cells on MSCs for bone regeneration are presented. The immune regulation of MSCs to immune cells is also described. Potential modulations are reviewed in detail to be used in bone tissue engineering by targeting the interaction between MSCs and immune cells. The main purpose of this review is to interpret the interaction between MSCs and immune cells coupled with potential modulation strategies. This review can be used as a basis for conducting future studies on bone tissue engineering that consider immunomodulation into bone regeneration.

## 2. Osteoinductive Effects of Immune Cells on MSCs for Bone Regeneration

Inflammation is an important part of bone regeneration. Immune cells create a suitable immune microenvironment for bone regeneration through phagocytosis, degranulation, and cytokine secretion. They also accelerate bone healing. MSCs are important precursor cells for fracture repair because they can differentiate into bone cells. Inflammatory stimulation can recruit MSCs, regulate the proliferation and apoptosis of MSCs, and promote their osteogenic differentiation. In this part, we mainly present the regulation and mechanism of immune cells on MSCs.

### 2.1. Recruitment

When bone tissue is damaged, neutrophils, macrophages, lymphocytes, and other immune cells first enter the bone regenerative microenvironment; in the early inflammatory microenvironment, immune cells mobilize MSCs by releasing soluble mediators, such as cytokines and chemokines; recruit them to the bone injury site; and differentiate into osteoblasts for bone repair [15]. MSCs express various chemokine receptors, such as C-C chemokine receptor type 1 (CCR1), CCR7, C-X-C chemokine receptor type 4 (CXCR4), CXCR5, and related chemokines, can act on these receptors and promote the migration of MSCs to the injured tissue [16, 17]. Many soluble factors can promote the migration of MSCs, including but not limited to stromal derived factor 1 (SDF-1) [18, 19], monocyte chemoattractant protein-1 (MCP-1) [20], macrophage inflammatory protein 1 (MIP-1) [21], RANTES (CCL5) [22], bone morphogenetic protein 2 (BMP-2) [23, 24], vascular endothelial growth factor (VEGF) [23], tumor necrosis factor alpha (TNF-*α*) [25, 26], and transforming growth factor-beta 3 (TGF-*β*3) [27]. Evidence has shown that SDF-1 is one of the mostly researched chemokines, which have the greatest influence on the migration of MSCs. It can act on CXCR4 on the surface of MSCs and induce MSCs to migrate to the bone injury site. Therefore, SDF-1 can even promote the proliferation and differentiation of MSCs [16, 19, 28].

Systemic or local inflammatory responses mediated by immune cells can effectively regulate the homing and recruitment of MSCs, which are essential for bone tissue regeneration [25]. In a low inflammation state, the chemotaxis and proliferation rate of MSCs decrease significantly, indicating that an inflammatory microenvironment plays an indispensable role in bone tissue regeneration. Macrophages secrete MCP-1 and MIP-1 at the inflammatory stage; consequently, they promote MSC migration through the extracellular regulated protein kinase (ERK) signaling pathway and induce the osteogenic differentiation of MSCs at the repair stage [29, 30]. In a macrophage-conditioned medium, the gene expression and cytokine secretion profile of MSCs change into a proinflammatory phenotype, and activated MSCs increase the secretion of interleukin-6 (IL-6), C-X-C chemokine ligand type 10 (CXCL10), and C-C chemokine ligand type 5 (CCL5) [22]. CCL5, CCL2, and IL-8 secreted by macrophages can promote the migration of MSCs to bone injury sites, and these soluble cytokines play a role by activating the stress-activated protein kinase/c-Jun N-terminal kinase (SAPK/JNK) signal pathway [22, 31]. T cells stimulated by TNF-*α* activate the nuclear factor-kappa B (NF-*κ*B) pathway and secrete more CCL5 to recruit MSCs to the injured site, thereby achieving the effect of ectopic osteogenesis [32]. Ponte et al. [25] studied the effects of 16 chemokines and growth factors on the migration of MSCs in vitro and agreed that TNF and IL-1*β* can cause MSCs to express more chemokine receptors; consequently, their sensitivity to chemokines improves. In their experiment, depleted T cells significantly reduce the infiltration of MSCs, confirming the recruitment effect of T cells on MSCs [33]. Further studies should explore the role of NK cells in bone tissue regeneration. NK cells can indirectly eliminate necrotic tissue and secrete neutrophil activating protein 2 (NAP-2) and RANTES to promote MSC migration [34]. After understanding the mechanism of immune cells recruiting MSCs, we can regulate the migration of MSCs by various means (biophysical and biochemical stimulation) to shorten fracture healing.

### 2.2. Proliferation and Apoptosis

Immune cell and the soluble factor secretion not only attracts MSCs to the site of bone injury but also regulates the survival of MSCs. Previous studies showed that M1 macrophages and their related cytokines (TNF-*α*, IL-1*β*,1L-6, and IFN-*γ*) inhibit the proliferation of MSCs. Although the use of TNF-*α* alone can increase the number of MSCs, its protective effect is insufficient to counteract the negative effects of other inflammatory cytokines. M2 macrophages and their related cytokines (IL-10, TGF- *β* 1, TGF-*β*3, and VEGF) promote the proliferation of MSCs, but the number of MSCs does not increase significantly when IL-10 is used alone [35, 36]. T lymphocytes can differentiate into many subtypes, and each subtype has different regulatory effects on MSCs. CD8^+^ T cells can partially inhibit MSC-mediated bone regeneration, CD4^+^ T cells completely inhibit osteogenic differentiation, and regulatory T cell (Treg) infusion can eliminate the inhibition of activated T cells on MSCs [37, 38]. Liu et al. [37] showed that activated T cells downregulate osteogenesis in a dose-dependent manner by secreting interferon-gamma (IFN-*γ*), and MSC apoptosis is induced by TNF-*α* secretion. Treg cells can resist this negative effect because it can reduce the expression of INF-*γ* and TNF-*α* [39, 40]. Aspirin, a nonsteroidal anti-inflammatory drug, also elicits this effect. Their experiments have also pointed out that IFN-*γ* mediates a nonapoptotic pathway of the downregulation of osteogenesis by activating Smad-6 in MSCs. With the cooperation of IFN-*γ*, TNF-*α* aggregates and internalizes Fas in MSCs, activates caspase8 and caspase3, and promotes the conversion of Fas nonapoptotic signals into apoptotic cascades [37]. However, some reports have suggested that activated T cells induce MSC apoptosis through Fas/FasL and CD40/CD40L pathways [41–44].

Previous studies provided different views on the role of TNF in mediating the proliferation and apoptosis of MSCs. Some scholars believed that TNF-*α* promotes the proliferation of MSCs, but opponents stated that TNF-*α* cooperates with INF-*γ* to induce MSC apoptosis. Two studies have reasonably explained this phenomenon. In particular, TNF-*α*, a member of the TNF family, can bind to tumor necrosis factor receptor 1 (TNFR1) and TNFR2 receptors and exert two main effects upon binding, namely, mediating programmed cell death via the Fas pathway and maintaining cell growth by activating the transcription factors NF-*κ*B, activator protein 1 (AP-1), and mitogen-activated protein kinase (MAPK) [45, 46]. In innate immune responses, activated neutrophils may induce MSCs to differentiate into osteoblasts by changing IL-1*α* and TGF-*β* levels [47]. Some experiments have also shown that neutrophils inhibit the production of the extracellular matrix from MSCs and even induce MSC apoptosis by producing reactive oxygen species (ROS) [48, 49].

### 2.3. Osteogenic Differentiation

The osteogenic differentiation of MSCs is the most critical part of bone regeneration, and the balance and dynamics between pro- and anti-inflammatory signals generated by immune cells are important for the osteogenic differentiation of MSCs. Bone morphogenetic proteins (BMPs) and Wnt signaling are two important signaling pathways known to regulate osteogenesis [14]. BMPs can promote osteogenesis by activating Smad protein and upregulating the Runt-related transcriptional factor 2 (Runx-2) expression [50]. The Wnt pathway regulates the osteogenic differentiation of MSCs through LDL receptor-related protein 5/6 (LRP5/6) [51]. Many immune cells and their cytokines regulate osteogenesis through these two pathways. For example, macrophage secretion of BMP-2 and TGF-*β* activates the corresponding Smad proteins, respectively, prompting downstream signaling molecules to translocate to the nucleus and upregulate the expression of Runx-2 and alkaline phosphatase (ALP), thereby promoting osteogenesis [52]. In addition, M1 macrophage secreted oncostatin M (OSM) promotes osteogenic differentiation and matrix mineralization of MSCs by activating the signal transducer and activator of transcription 3 (STAT3) pathway [53]. IL-1*β* promotes osteogenic differentiation of MSCs through noncanonical Wnt-5a and receptor tyrosine kinase-like orphan receptor 2 (Ror2) signaling pathways [54]. However, there are not many reports on the signaling pathways by which immune cells promote osteogenic differentiation of MSCs, which may be a direction for subsequent studies.

In innate immune cells, the monocyte–macrophage line promotes the osteogenic differentiation of MSCs by releasing BMP-2 and TGF-*β*1, and human monocytes promote the expression of the osteogenic signals of MSCs, such as Runx-2, BMP-2, and ALP [52]. Although macrophages have been recognized to be involved in MSC-mediated bone regeneration, the phenotype most conducive to osteogenesis remains unclear. Most investigators believed that M1 macrophages secrete the proinflammatory cytokines TNF-*α* and IL-1*β* to reduce the expression levels of Runx-2 and ALP, thereby hurting the osteogenic differentiation of MSCs [55–57]. This partial mechanism is feasible for bone loss in rheumatoid arthritis and postmenopausal osteoporosis although bone loss is often regarded as a result of increased osteoclast activities [36, 58]. The positive effect of M2 macrophages on the osteogenic differentiation of MSCs is undeniable. Chen et al.'s team produced *β*-tricalcium phosphate-coated magnesium scaffolds (Mg-*β*-TCP scaffolds) that polarize macrophages to the M2 phenotype, and M2 macrophages secrete BMP-2 and VEGF in synergy with each other to promote osteogenic differentiation and angiogenesis in MSCs [59]. The biomimetic hierarchical intrafibrillarly mineralized collagen (HIMC) scaffold promotes macrophage polarization and enhances the osteogenic properties and mineralization potential of MSCs mainly through IL-4, which seems to be detrimental to the recruitment of MSCs; furthermore, this effect of IL-10 on MSCs is negated in their experiments [60]. However, some new ideas have described that all phenotypes of macrophages can promote ALP gene expression and matrix mineralization, even demonstrating that the osteoinductive effect of M1 is the strongest of all subtypes [61, 62]. When cocultured with MSCs, unpolarized macrophages promote osteogenic differentiation by inducing the expression of OSM [63, 64]. This factor induces the osteogenic differentiation of MSCs and promotes matrix mineralization through the STAT3 pathway [53, 65]. OSM synergizes with TNF-*α* to upregulate ALP activity and promote bone regeneration [61]. Lu et al. [66] demonstrated that proinflammatory M1 macrophages promote the MSC-mediated increase in bone mass via the prostaglandin E2 (PGE2) pathway.

In adaptive immune cells, T cells can inhibit osteogenesis in homozygous MSCs; by contrast, homozygous MSCs are induced in Rag1−/− mice lacking T cells [67]. The bone regeneration of T cell-deficient mice strengthens with an increased gene expression of BMP-2, bone sialoprotein (BSP), and type II collagen [68]. However, T cell deficiency is detrimental to bone regeneration, and the proinflammatory cytokine IL-17F secreted by TH17 cells positively affects osteogenic differentiation [69]. Ono et al. [70] showed that IL-17A secreted by T cells shifts the lipogenic differentiation capacity of MSCs toward osteogenic differentiation, thereby increasing the quality of bone healing. However, generalizing the role of T or B cells in bone regeneration is not rigorous enough because lymphocytes have many subtypes, and each subtype has a different role in osteogenesis. The “synergistic effect” of each subtype on MSCs may be biased in different microenvironments, so different perspectives have been provided. Further studies are needed to elucidate the role of different T cell subtypes in bone healing. TH1 immune response has been reported to inhibit osteogenic differentiation, and IFN-*γ* plays a major role in this process because IFN-*γ* downregulates the expression of Runx-2, osteocalcin (OCN), and ALP [37, 67]. On the contrary, Treg cells can suppress proinflammatory T cells and even participate directly in osteogenic differentiation and promote bone regeneration. In addition, the role of B lymphocytes in bone regeneration has been less reported and is mainly considered to be related to the function of osteoclasts. Activated B cells increase the activity and number of osteoclasts through the release of receptor activator for nuclear factor kappa B ligand (RANKL) and promote bone resorption. However, studies should be performed to determine whether B cells play a dominant role in bone regeneration and reveal the mechanism of their activities.

**Figure 1:**
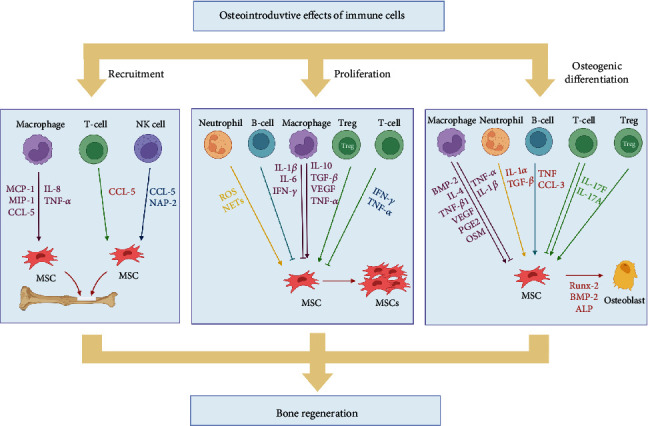
Schematic illustration of the regulation of mesenchymal stem cells (MSCs) by immune cells in bone regeneration. The positive effect of immune cells on MSCs is important for bone regeneration. Immune cells can promote or inhibit the recruitment, proliferation, and osteogenic differentiation of MSCs. Soluble factors secreted by immune cells play an important role in this process. Abbreviations: MCP-1: monocyte chemoattractant protein-1; MIP-1: macrophage inflammatory protein 1; CCL5: C-C chemokine ligand type 5; IL-8: interleukin-8; TNF-*α*: tumor necrosis factor alpha; NAP-2: neutrophil activating protein 2; ROS: reactive oxygen species; NETs: neutrophil extracellular traps; IFN-*γ*: interferon-gamma; TGF-*β*: transforming growth factor-beta; VEGF: vascular endothelial growth factor; BMP-2: bone morphogenetic protein 2; Runx-2: Runt-related transcriptional factor 2; PGE2: prostaglandin E2; OSM: oncostatin M; ALP: alkaline phosphatase; Tregs: regulatory T cells. Created with BioRender.com.

The most important mediator of osteogenesis regulation by immune cells is cytokines; as such, many studies have been conducted on the role of cytokines, but the role of various cytokines in osteogenic differentiation is still uncertain. For example, TNF-*α* has been reported to favor the recruitment and proliferation of MSCs and promote osteogenic differentiation and matrix mineralization through the upregulation of Runx-2, OCN, and ALP at low doses and brief exposure [71, 72]. However, at high doses, TNF-*α* inhibits osteogenic differentiation through the downregulation of insulin-like growth factor 1 (IGF-1), Runx-2, and Osx expression [73–75]. Other cytokines (e.g., IFN-*γ*, TGF-*β*, and IL-1) have been reported to have such contradictory effects on bone regeneration, which may be related to the concentration of various cytokine effects, the duration of exposure, and the receptors on which they act. More definite effects should be explored in further studies (Figure 1).

## 3. Immune Regulation of MSCs to Immune Cells

MSCs are multipotent stem cells that have immunomodulatory functions and anti-inflammatory capabilities, in addition to differentiating into various lineages of cells. MSCs can modulate the proliferation and phenotype of T lymphocytes and macrophages, prevent B lymphocytes from secreting antibodies, inhibit DC maturation, and attenuate NK cell cytotoxicity. Moreover, immune regulation by MSCs is plastic. Combined with the role of immune cells on MSCs in osteogenesis, we venture to speculate that the crosstalk between MSCs and immune cells has a positive feedback-like effect on the regulation of bone regeneration. Therefore, in this part, we review the immunomodulatory effects of MSCs. Stem cell therapies can be better applied by exploring the potential mechanisms of immune regulation.

### 3.1. T Cells

T lymphocytes are derived from bone marrow hematopoietic stem cells and widely distributed in all tissues of the body [76]. The activation of naïve T lymphocytes requires stimulation by T cell receptors and costimulatory signals, and activated T cells differentiate into various subtypes depending on cytokines in the microenvironment [77]. Activated CD4^+^ T cells mainly differentiate into four subsets: TH1, TH2, TH17, and Treg. Among them, the differentiation of TH1 cells is mainly induced by cytokines such as IL-2, IL-12, and IFN-*γ*, and its main downstream effectors are IFN-*γ* and TNF-*α*. The main role of TH1 is to recruit macrophages and induce B cells to produce IgG. TH2 differentiation is mainly guided by IL-2 and IL-4, and its following effectors are IL-4, IL-5, and IL-13. The main role of TH2 is to recruit eosinophils and mast cells and induce IgE antibody production by B cells [78, 79]. TH17 is a special type of T-helper cells, which can accumulate at the site of bone injury and enhance the body's immune response under the action of chemokines such as CCL2 and CCL20 secreted by neutrophils. IL-17 expressed by activated TH17 can activate dendritic cells to release granulocyto-colony-stimulating factor (G-CSF) and induce a large number of neutrophils to migrate to the site of injury, resulting in a sustained inflammatory response. Tregs suppress excessive autoimmune responses and play an important role in bone regeneration. In the specific inflammatory microenvironment, CD8^+^ T cells differentiate into cytotoxic T lymphocytes (CTLs), which produce the proinflammatory cytokine IFN-*γ* that effectively kills infected target cells by releasing granzyme and perforin [80].

The interaction between MSCs and T cells has been widely studied. Previous studies showed [81–83] that MSCs inhibit the proliferation of effector T cells and induce the production of regulatory T cells mainly through direct cell–cell contact [84, 85] and soluble cytokine secretion [86, 87]. The soluble factors involved in the immunomodulatory effects of MSCs include IL-10 [88, 89], TGF-*β*1 [84, 90], nitric oxide (NO), indoleamine 2,3-dioxygenase (IDO) [91], PGE2 [92, 93], and TNF-*α*-stimulated gene 6 (TSG-6) [94, 95].

MSCs can affect the proliferation and apoptosis of T cells. MSCs elicit inhibitory effects on T cell proliferation in several experimental models. In a model of graft-versus-host disease, graft-derived MSCs strongly inhibit the proliferation of host T cells in vivo [82]. However, the mechanism of MSC-mediated suppression of T cell proliferation is uncertain. In a coculture assay, MSCs block the mitotic cycle of T cells in the G1 phase, where the complete blockage of intracellular DNA synthesis is detected, by releasing large amounts of TGF-*β*, downregulating cyclin D2, and upregulating p27^kip1^ [83]. Moreover, when the suppression of T cells by MSCs is removed in the experiment and a strong stimulation (IL-2) is applied, although T cell activity is restored, T cell proliferation is irreversible, fully demonstrating the dominant role of TGF-*β* in restraining T cell proliferation by MSCs. Park et al. [90] transferred the adenoviral TGF-*β* gene into MSCs, which produce a stronger immunosuppression than normal MSCs, further demonstrating the important role of TGF-*β* in this process. Although a previous study was that MSCs only block T cell proliferation and do not lead to T cell regulation [96], however, an increasing number of studies have reported that MSCs also induce T cell death through multiple pathways [81, 97]. MSCs can mediate the regulation of activated T cells via the FasL/Fas pathway [97], and FasL is necessary for the immunotherapeutic effect of MSCs in the treatment of mice with colitis [85]. FasL, a member of the TNF family, can induce apoptosis through a direct contact with the cell surface receptor Fas. Activated T cells are more sensitive to FasL-mediated apoptosis because of their high Fas expression [98]. Human placenta-derived mesenchymal stem cells (hpMSCs) also mediate Th1 and Th2 death via the immunosuppressive molecules programmed cell death protein 1 (PD-1) and Galectin 9 (Gal-9) [82, 99]. In addition, IDO is the rate-limiting enzyme for tryptophan catabolism, and a large amount of tryptophan is catabolized to kynurenine upon IDO overexpression [100]. Kynurenine causes the death of activated T cells [101]. Thus, IDO is one of the pathways through which MSCs regulate immunity [91, 102]. Experimental evidence has shown that MSCs need IFN-*γ* stimulation for IDO expression, which explains the inability of MSCs to induce the regulation of inactivated T cells [103, 104].

MSCs also regulate the activation and differentiation of T cells. Previous studies demonstrated that Th1 and Th17 are associated with the development of most autoimmune diseases [105, 106], whereas Th2 and Treg cells have a protective effect [107, 108]. MSCs inhibit the differentiation of naïve CD4^+^ T cells to Th1 and Th17 cells and promote their differentiation into Th2 and Treg cells. The infusion of human adipose-derived mesenchymal stem cells (hASCs) suppresses the number of Th1 and Th17 cells and increases CD4^+^CD25^+^Foxp3^+^ Treg cells, thereby significantly reducing bone and cartilage damage in arthritic mice [109]. Diverse opinions are available on the mechanism of Treg cell production induced by MSCs; according to the common concept, MSCs act through soluble cytokines [84], such as PGE2, IL-10, TGF-*β*1, and Notch signaling pathways [110]. When MSCs are added during the induction of CD4^+^ T cell differentiation, the expression levels of PGE2 and TGF-*β* significantly increase, and MSC-mediated immunosuppression can be reversed after treatment with the corresponding monoclonal antibodies [111]. This experiment also proposes that the Treg produced by MSCs cocultured with CD4^+^ T cells is an induced Treg (iTreg), not a proliferation of natural Tregs (nTregs) in the thymus. In addition, IL-10 is widely recognized to be involved in MSC-mediated immunosuppression. IL-10 inhibits T cell proliferation and promotes Treg cell differentiation by interacting with the histocompatibility leukocyte antigen G5 (HLA-G5), and HLA-G5 must be involved in promoting Treg differentiation by using cell contact [89]. In a previous study, MSCs are separately cocultured with each purified T cell subtype, and results demonstrate that the activation of T cells and their cytokine release are regulated by MSCs for each subtype [93].

The immunomodulation of MSCs is plastic. MSCs not only elicit immunosuppressive effects but also promote immune responses under specific circumstances, which is the plasticity of MSC immunomodulation [112]. This plasticity is mainly determined by the inflammation level in the microenvironment. In acute inflammatory responses, high levels of local inflammatory factors induce MSCs to transform to an immunosuppressive phenotype, which releases large amounts of immunosuppressive factors and suppresses the immune response; conversely, in chronic inflammatory responses, a local microenvironment induces MSCs to transform to a proinflammatory phenotype, and they release chemokines at the injury site to recruit more immune cells, which cannot be suppressed, thereby exacerbating the immune response [113]. Li et al. [114] investigated the effect of different INF-*γ* concentrations on the immunomodulatory effect of MSCs. Low IFN-*γ* concentrations can stimulate only a small amount of NO production in mouse MSCs; when the IFN-*γ* concentration increases, it can produce enough NO to elicit immunosuppressive effects on MSCs, fully demonstrating this plasticity.

### 3.2. Macrophages

Macrophages differentiate from hematopoietic stem cells and act as the main innate immune cells that classically serve to engulf nonautologous materials (e.g., bacteria, viruses, and grafts) and necrotic tissues. They also expose their antigens for presentation to T lymphocytes. Thus, they have a fundamental role in the body's immune defense. Macrophages have significant plasticity, and undifferentiated M0 macrophages can polarize into two major phenotypes under certain conditions: M1 and M2. This process of polarization into different phenotypes depends on the local microenvironment of injury sites. M1 macrophages are proinflammatory phenotypes and initial response cells to injury or foreign body stimulation. M0 macrophages can differentiate into M1 macrophages under the influence of cytokines such as INF-*γ* and TGF-*α*. In turn, these cytokines can secrete TNF-*α*, IL-6, IL-1*β*, IL-12, and other inflammatory factors to induce local inflammatory responses and promote the differentiation of macrophages into osteoclasts. As a result, bones undergo inflammatory resorption. Similarly, M0 macrophages differentiate into M2 macrophages under the induction of cytokines such as IL-4 and IL-13. M2 macrophages are anti-inflammatory phenotypes that can secrete biological mediators such as IL-10, TGF-*β*1, VEGF, BMP-2/4, and CCL-13/18 to promote inflammation regression and tissue reconstruction [115–117].

Macrophages play an important role in bone tissue regeneration, and MSCs can further promote fracture repair through their regulation. MSCs can secrete soluble factors such as PGE2 [118], interleukin-1 receptor antagonist (IL-1RA)[119], TSG-6 [94], TGF-*β*1 [120], IL-6 [121], and IDO [122], which induce the conversion of M1 macrophages to M2 macrophages, thereby suppressing systemic and local inflammatory responses and promoting bone tissue regeneration and wound repair. A coculture assay shows that MSCs induce polarization from M1 macrophages to M2 macrophages by secreting PGE2, which downregulates the expression of the proinflammatory cytokines TNF-*α*, IL-1, and IL-6 and upregulates the expression of the anti-inflammatory factor IL-10; consequently, local inflammatory responses decrease, and a suitable microenvironment for bone regeneration forms [118]. In addition, MSCs upregulate IL-10 expression and attenuate systemic sepsis by reprogramming macrophage polarization through PGE2 [123]. Further studies have revealed that MSCs promote PEG2 secretion by upregulating caspase activity and activating NF-*κ*B; PGE2 acts on E-prostanoid receptor 4 (EP4) on the surface of macrophages to induce their polarization [124]. In addition to PGE2, IL-1RA is involved in macrophage polarization, and MSCs lacking IL-1RA are less capable of inducing M1 to M2 conversion than those in wild-type mice [119, 125]. In a mouse model of sepsis, TGF-*β*1 secreted by MSCs then mediates the altered macrophage phenotype through the activation of the AKT/FoxO1 signaling pathway [126]. In coculture with monocyte experiments, MSCs secrete IDO to induce the differentiation of monocytes to M2 directly, demonstrating that M2 can indirectly inhibit T cell proliferation [127]. In addition to cytokines, the metabolites of MSCs appear to play a role in MSC immunomodulation; evidence has suggested that lactate produced by MSCs alters mitochondrial activity through metabolic reprogramming, thereby tilting monocytes toward M2 differentiation [128]. MSCs can secrete CCL2 and CCL4 to recruit circulating macrophages and vascular endothelial cells to the bone injury site, which can then be regulated to create favorable conditions for osteogenesis in MSCs [129, 130]. Cell contact and inflammatory factor stimulation likely enhance the immunosuppressive function of MSCs, which can be confirmed in the Transwell system and IFN-*γ* or lipopolysaccharide- (LPS-) pretreated MSC models [120, 131].

MSCs can regulate macrophage differentiation into a Treg-like regulatory macrophage by secreting PGE2, which has a cytokine secretion profile different from that of classical anti-inflammatory M2 macrophages, expressing high IL-10 and IL-6 levels and low IL-12 and TNF-*α* levels [118, 131]. Although such regulatory macrophages express high levels of IL-6, endogenous IL-6 secreted by macrophages has significant anti-inflammatory and tissue repair-promoting effects; thus, the phagocytic capacity is enhanced [132, 133].

### 3.3. B Cells

B lymphocytes are important cells in the adaptive immune system. Surface receptors on naïve B cells recognize specific antigens, proliferate, and differentiate into plasma cells and memory cells that can produce antibodies to clear pathogens and protect organisms [134]. The mesenchymal stem cells of different origins inhibit B cell proliferation, differentiation, and chemotaxis in vivo and in vitro [135]. In a systemic lupus erythematosus (SLE) mouse model, MSCs significantly inhibit the ability of activated B cells to proliferate and differentiate to plasma cells while increasing B cell activities [136]. MSCs inhibit B cell proliferation by blocking the cell cycle in the G0/G1 phase without inducing the apoptosis of B and plasma cells [135], and human adipose-derived MSCs even contribute to the survival of inactivated B cells [137, 138]. However, the mechanism by which MSCs inhibit B cells is unclear. On the one hand, MSCs exert their regulatory effects on B cells by upregulating ERK1/2 and inhibiting p38MAPK phosphorylation [139]. Asari et al.[138] and Che [140] demonstrated that MSCs inhibit the differentiation of B cells into plasma cells by downregulating Blimp-1 expression and upregulating paired box protein 5 (PAX-5) expression, thereby reducing IgG and IgM secretion. Transcription factors such as Blimp-1, PAX-5, B cell lymphoma 6 (BCL-6), and X-box binding protein 1 (XBP1) are the main regulators of the differentiation of B cells into plasma cells [141–144]. On the other hand, MSCs induce the differentiation of B cells to regulatory B cells (Bregs), which can secrete large amounts of IL-10 and negatively affect immune responses [119, 137]. As for T cells, stimulation by inflammatory factors enhances the suppressive effect of MSCs on B cells. Corcione et al. [135] reported that MSCs require B cell paracrine factor activation to function because the supernatant of MSCs in culture alone does not exert an inhibitory effect. Subsequent studies have also demonstrated an overarching role for IFN-*γ* [145]. In addition, intercellular contacts are involved in this process and may be related to the role of PD-1 and programmed cell death ligand 1 (PD-L1) [145]. Several conflicting results have been reported; in particular, few researchers showed that coculture with MSCs promotes B cell proliferation, differentiation, and antibody secretion [146, 147]. These variations may reflect differences in MSC source, B cell proliferation status, stimulation intensity, purification process, and experimental methods. MSCs induce more IgG production in B cells under weak stimulation [135]. Therefore, MSCs inhibit the proliferation of B cells in a concentration-dependent manner [138, 140].

### 3.4. NK Cells

NK cells are innate immune cells that exert their immune effects through the release of cytokines IFN-*γ*, TNF-*α*, and IL-10 upon activation. They are cytotoxic, releasing granzyme and perforin through degranulation to kill virus-infected cells and tumor cells [148, 149]. They also participate in bone tissue regeneration. In comparison with T cells and macrophages, few current studies have been performed on the regulation of NK cells by MSCs. MSCs are potent inhibitors of NK cells because they can inhibit NK cell proliferation, cytokine secretion, and cytotoxicity in an inflammatory environment [150, 151]. MSCs inhibit the IL-15-stimulated proliferation of NK cells and the secretion of cytokines such as IFN-*γ* by secreting soluble factors; MSCs reduce the cytotoxicity of NK cells in a cell-to-cell contact manner, but they did not induce NK cell death [151]. The inhibitory effect of MSCs on NK cells can be completely reversed by adding the neutralizing factors of TGF-*β*1 and PGE2, suggesting that TGF-*β*1 and PGE2 may be the two main mediators of the effect of MSCs [151]. NK cell-derived IFN-*γ* can promote the expression of IDO by MSCs, thereby inducing immunosuppression [150]. The regulation of NK cells by MSCs is also related to many factors. On the one hand, it is dose dependent, and the inhibition of NK cell cytokine secretion by MSCs is related to the NK/MSC ratio, with a significant inhibitory effect only when the NK/MSC ratio is low [151]. On the other hand, it is time dependent, and MSCs can promote NK cell activation in early stages and inhibit their activity in late stages; this phenomenon may be related to phenotypic changes in MSCs [152]. However, some contradictory results have suggested that MSCs can promote the secretion of cytokines such as IFN-*γ* and TNF-*α* by NK cells and upregulate the degranulation of NK cells; consequently, a stronger inflammatory response is induced [153]. These paradoxical results may be attributed to factors such as dose and time and related to the source of MSCs or NK cell subpopulation; however, they should be further confirmed in future studies.

### 3.5. Dendritic Cells

Dendritic cells (DCs) are the most powerful specialized antigen-presenting cells (APCs) in the body. Mature DCs can efficiently extract and process antigens and present them to T lymphocytes to initiate an adaptive immune response [154, 155]. MSCs equally can exert their immunomodulatory effects by suppressing DCs. Similar to the mechanism by which MSCs inhibit T cell proliferation, MSCs inhibit the differentiation of monocytes into DCs through the downregulation of cyclin D2 expression, which blocks the cell cycle in the G0 phase [156]. MSCs also block DC maturation through a few other pathways. Among them, miRNAs play a key role in regulating DC maturation, and MSCs can inhibit DC maturation by upregulating the expression of miR-23b and blocking the activation of the NF-*κ*B pathway [157]. IL-6 and macrophage-stimulating factor (M-CSF) act synergistically in DCs to interfere with their maturation [158]. However, Spaggiari et al. suggested that PGE2 plays a dominant role in the inhibition of DC maturation in MSCs independent of IL-6 [159]. Moreover, PD-L1, NO, and adenosine may be involved in this process, and IFN-*γ*-activated MSCs elicit stronger inhibitory effects [160]. Interestingly, van den Berk et al. noted that cord blood-derived mesenchymal stem cells (USSCs) act more differently than bone marrow-derived mesenchymal stem cells (BM-MSCs) because they positively affect DC maturation and function [161]. This finding implies that our subsequent studies should consider cell sources. Furthermore, immature DCs (iDCs) can suppress T cell responses and induce antigen-specific tolerance; therefore, the suppression of T cell-mediated immune responses and Treg cell production by MSCs may be associated with impaired DC maturation [162].

### 3.6. Neutrophils

Neutrophils are potential immune cells that enter the injury site and serve as the first line of defense of the body's immunity [163]. During the inflammatory response phase of fracture repair, neutrophils remove necrotic tissues and defend against pathogenic invasion through phagocytosis, cytokine secretion, and neutrophil extracellular trap (NET) production, providing a suitable local microenvironment for bone regeneration and initiating fracture repair. MSCs exhibit inhibitory effects on most immune cells; however, they positively affect neutrophils. MSCs inhibit neutrophil regulation, alter their chemotaxis, and enhance respiratory burst capacity [164–166]. MSCs prolong the lifespan of neutrophils even at low relative MSC concentrations. Moreover, upregulated myeloid cell leukemia-1 (MCL-1) and downregulated Bax are detected in the culture system as a result of the activation of the STAT3 signaling pathway by MSC-derived IL-6 [164]. Although inactivated MSCs elicit this antiapoptotic effect, MSCs with activated Toll-like receptors (TLRs) have a stronger benefit, and IL-6, IFN-*γ*, and granulocyte-macrophage colony-stimulating factor (GM-CSF) produced by activated MSCs act cooperatively to retard neutrophil death [167]. In addition, MSCs secrete IL-8 and macrophage migration inhibitory factor (MIF) to recruit neutrophils and facilitate their entry into inflammation sites; the recruited neutrophils exhibit more vigorous antimicrobial activity and respiratory burst capacity [165, 168]. Interestingly, in a mouse model of vasculitis, MSCs inhibit NET production and uncontrolled respiratory bursts by overactivated neutrophils and effectively reduce the release of oxygen radicals from neutrophils by secreting the antioxidant enzyme superoxide dismutase 3 (SOD3) [169]. For the first time, Jiang et al. [169] demonstrated that MSCs can phagocytose overactivated neutrophils, reducing the tissue damage caused by the release of toxic particles from neutrophils. In a corneal injury model and a peritonitis mouse model, TSG-6 secreted by MSCs effectively inhibits the entry of neutrophils into the injury site, thereby significantly reducing inflammatory responses [94, 95]. Such a biphasic effect indicates the plasticity of immune regulation in MSCs, and we speculate whether MSCs can trigger the necessary inflammatory response while suppressing excessive immune responses and maintaining a local microenvironment conducive to tissue repair.

**Figure 2:**
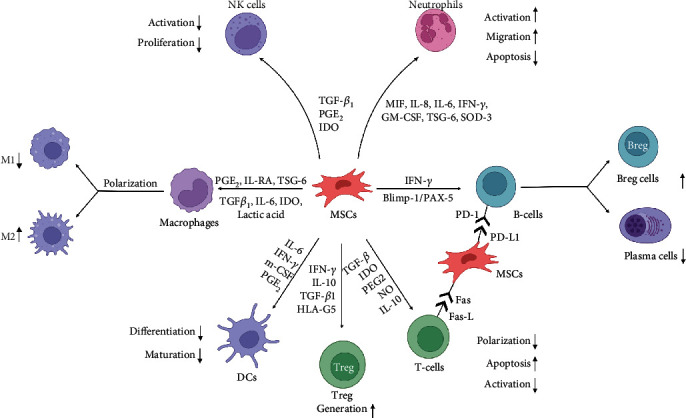
The complex mechanisms of immunomodulatory functions of MSCs. Both the innate and adaptive immune systems are regulated by MSCs. MSCs can modulate the maturation and activation, proliferation and regulation, migration, and differentiation of immune cells. In addition, MSCs can also regulate the polarization of macrophages and promote Treg cell production. The main mechanisms by which MSCs exert immunomodulatory effects are secretion of soluble factors and direct cell-cell contacts. The potential implications of intercellular contacts are interactions of PD-1/PD-L1 and Fas/Fas-L. Abbreviations: IL-1RA: interleukin-1 receptor antagonist; IDO: indoleamine 2,3-dioxygenase; TSG-6: TNF-*α*-stimulated gene 6; MIF: macrophage migration inhibitory factor; GM-CSF: granulocyte-macrophage colony-stimulating factor; M-CSF: macrophage-stimulating factor; SOD3: superoxide dismutase 3; PAX-5: paired box protein 5; HLA-G5: histocompatibility leukocyte antigen G5; NO: nitric oxide; PD-1: programmed cell death protein 1; PD-L1: programmed cell death ligand 1; DCs: dendritic cells; Bregs: regulatory B cells. Created with BioRender.com.

In conclusion, the mechanisms of the immunomodulatory effects of MSCs are complex, and characterizing a single factor is difficult because of the interaction between various factors; thus, many paradoxical views on such mechanisms have been described. This phenomenon may also be related to different species of experimental animals, various tissue sources of MSCs, and variations in experimental design protocols. More in-depth studies are needed to fully understand and exploit the mechanisms of immunomodulation by MSCs (Figure 2).

## 4. Modulations of the MSC–Immune Cell Interaction for Bone Tissue Engineering

**Figure 3:**
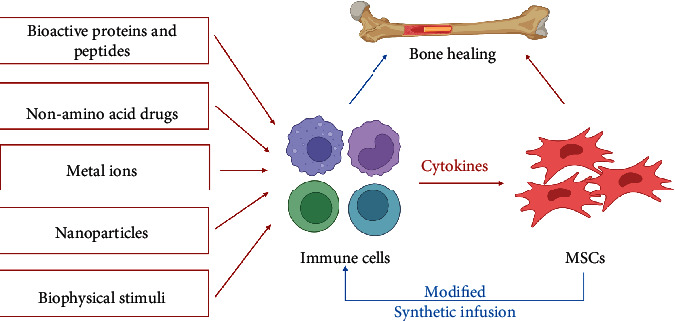
The modulations of immune cells to affect MSCs for bone tissue engineering. Biochemical stimuli (bioactive proteins or peptides, nonamino acid drugs, metal ions, and micro- and nanoparticles) and biophysical stimuli (internal structural stimuli, external mechanical stimuli, and electromagnetic stimuli) could modulate immune cells to affect MSCs for bone healing. The loading of modified MSCs and the systemic infusion of MSCs could regulate immune cells to promote bone healing. Created with BioRender.com.

In bone tissue engineering, MSCs can be incorporated into biomaterials by direct encapsulation or indirect recruitment. Although most studies have contributed to the direct stimulation of osteogenic MSC differentiation, modulating the interaction between MSCs and immune cells may be a novel approach for bone tissue engineering. The methodology can be divided into two orientations: modulations of immune cells to affect MSCs and modulations of MSCs to influence immune cells (Figure 3). Both orientations help provide a suitable microenvironment for bone regeneration.

### 4.1. Modulations of Immune Cells to Affect MSCs for Bone Tissue Engineering

Various cues modulate immune cells to affect MSCs, which can be categorized as biochemical stimuli (bioactive proteins or peptides, nonamino acid drugs, metal ions, and microparticles and nanoparticles) and biophysical stimuli (internal structural stimuli, external mechanical stimuli, and electromagnetic stimuli) [170].

#### 4.1.1. Bioactive Proteins and Peptides

Bioactive proteins and peptides elicit immunomodulatory effects on MSCs to promote osteogenesis; among them, cytokines have been widely used. IL-4 is one of the most used anti-inflammatory cytokines that can promote the anti-inflammatory M2 macrophage polarization, which can further facilitate bone regeneration. Hu et al. [171] introduced IL-4 into a heparin-modified gelatin microsphere, which can realize the controlled release of IL-4 because of the binding interaction between IL-4 and heparin. This binding can also stabilize IL-4 and prevent it from denaturation and degradation, ensuring the immunomodulatory activity of IL-4 to diminish local inflammation and promote bone neoformation [171]. Considering that the M1 phenotype initiates angiogenesis and the M2 phenotype stimulates vessel maturation, Spiller et al. [172] incorporated interferon-gamma (IFN-*γ*) in scaffolds by weak physical absorption and IL-4 by strong biotin-streptavidin binding; consequently, a short release of IFN-*γ* and a sustained release of IL-4 occur. The sequential polarization of M1 and M2 macrophages promotes vascularization [172]. Therefore, utilizing cytokines to promote the sequential activation of M1/M2 macrophage and a timely and successful transition from a pro-inflammatory M1 phenotype to the M2 phenotype may contribute to bone regeneration.

In addition to cytokines, some growth factors show immunomodulatory functions and osteoinductive effects. BMP-2 has been widely used for bone regeneration, and several approaches have been developed to reduce side effects by lowering therapeutic doses [173, 174]. BMP-2 also shows an immunomodulatory capacity [175]. Wei et al. [176] showed that BMP-2 can promote the infiltration and recruitment of macrophages and downregulate M1 phenotypic markers, including IL-1*β*, IL-6, and iNOS. Bioactive factors secreted by BMP-2-stimulated macrophages can promote MSC osteogenesis [176]. Furthermore, BMP-2 utilization can couple with other cytokines to enhance bone regeneration. Zou et al. [177] simultaneously loaded IL-4 and BMP-2 to graphene oxide (GO) for their controlled release and encapsulated into carboxymethyl chitosan (CMC)/poly(ethylene glycol) diacrylate (PEGDA) hydrogel. They showed that the hydrogel embedded with IL-4 and BMP-2 can dramatically promote the M2 polarization of macrophages and the osteogenic differentiation of MSCs. Hydrogel can also promote bone healing with a reduced inflammatory response. In addition to BMP-2, other growth factors from platelet-rich plasma (PRP), such as IGF, PDGF, and TGF-*β*, may improve M2 phenotype polarization, which then enhances bone regeneration [178].

In comparison with bioactive proteins, bioactive peptides may be more promising because of facile production and low cost. Cationic antimicrobial peptides are immunomodulatory peptides showing anti-infective and anti-inflammatory activities [179]. Chen et al. [180] immobilized GL13K, a cationic antimicrobial peptide, to the surface of titanium, which is seeded with M1 or M2 macrophages. They demonstrated that GL13K-modified titanium inhibits the M1 phenotype but suits the M2 phenotype with ideal cytocompatibility [180]. GL13K-modified titanium also reduces the expression of proinflammatory cytokines, including TNF-*α* and IL-1*β*, but it promotes the expression of anti-inflammatory cytokines, including IL-10 and TGF-*β*3, which may promote bone regeneration [180]. Besides, parathyroid hormone (PTH), one 84-amino acid peptide secreted from the parathyroid gland, is another immunomodulatory peptide for bone regeneration because PTH can promote T cells to express the Wnt ligand and activate the related Wnt signaling [181]. PTH and PTH-related peptides have been locally used in bone tissue engineering [182]. Future studies should focus on its immunomodulatory effects on bone healing. Other immunomodulatory peptides include calcitonin gene-related peptide (CGRP) [183] and alpha melanocyte-stimulating hormone [184], which show great potential for bone tissue engineering.

#### 4.1.2. Nonamino Acid Drugs

In recent years, some nonamino acid drugs possessing immunomodulatory functions have been used as potential alternatives to bioactive proteins and peptides in bone tissue engineering. Liu et al. [185] incorporated fingolimod (FTY720), an immunoregulator derived from myriocin, into a mesoporous bioactive glass, which can realize a controlled release for 7 days because of mesoporous properties and electrostatic binding. The released FTY720 can promote M2 macrophage polarization, which then improves osteogenesis and inhibits osteoclastogenesis [185]. Rosiglitazone (RSG) is a synthetic highly selective agonist of peroxisome proliferator-activated receptor-*γ* (PPAR*γ*), which is introduced to a xenogeneic decellularized matrix (xNDM) [186]. RSG can stimulate M2 macrophage polarization and antagonize M1 macrophage polarization with the evidence of improved IL-10 and TGF-*β* and decreased IL-1 and TNF-*α* [186]. RSG-embedded xNDM shows improved bone regeneration with higher osteogenic markers, including ALP, osteopontin (OPN), and dentin sialoprotein (DSP) [186]. Nonsteroidal anti-inflammatory drugs (NSAIDs) are also one of the promising immunomodulatory drugs for bone regeneration; among them, aspirin has been developed for bone tissue engineering, especially for inflammatory conditions [187]. In rat mandibular bone defects with inflammatory conditions, aspirin treatment dramatically enhances bone regeneration because aspirin can diminish macrophage activation induced by lipopolysaccharide (LPS); evidence has shown lowered inducible NO-synthase (iNOS) and TNF-*α* [188]. Moreover, some nonamino acid hormone drugs can be introduced to biomaterial scaffolds for bone regeneration. Estrogen is a hormone that can regulate the immune system [189]. It has been used in bone tissue engineering [190], but its immunomodulatory effects have not yet been explored. Dexamethasone is another promising hormone to be used for bone tissue engineering because it can inhibit inflammation.

#### 4.1.3. Metal Ions

The introduction of metal ions with an immunomodulatory bioactivity is another strategy to construct a suitable immune microenvironment for bone regeneration. Multiple metal ions show immunomodulatory effects; among them, Ca, Co, and Si ions promote inflammation, whereas Ca, Zn, Mg, and Sr ions diminish inflammation [191]. However, the toxicity of metal ions may be initiated when their concentrations are above the therapeutic dosage [192]. Therefore, the application of metal ions should be limited in a controlled release pattern. Song et al. [193] encapsulated zinc silicate into a nanohydroxyapatite/collagen scaffold, which can achieve the sustained release of silicate ions and Zn ions. Zinc silicate can activate and promote monocytes to differentiate to tartrate-resistant acid phosphatase- (TRAP-) positive cells; these cells then secrete prohealing factors (SDF-1, TGF-*β*1, VEGF-*α*, and PDGF-BB) to recruit MSCs and endothelial cells for bone regeneration [193]. Zhang et al. designed a strontium-substituted submicrometer bioactive glass (Sr-SBG), which elicits appropriate immunomodulatory effects. The released Sr ions from scaffolds can promote M2 macrophage polarization, consequently improving the M2 marker CD206. Immunomodulatory scaffolds also downregulate inflammatory genes (IL-1*β* and iNOS) and upregulated anti-inflammatory genes (IL-1*γα* and arginase). An in vivo study has demonstrated that Sr-SBG promotes more bone formation with a less detrimental immune response than SBG without strontium.

#### 4.1.4. Nanoparticles

Nanoparticles can be used as immunoregulators to be encapsulated in biomaterials. Nanohydroxyapatite (HA) particles promote M2 macrophage polarization, while micron-sized HA particles promote M1 macrophage polarization [194, 195]. In comparison with micron-sized HA particle-encapsulated scaffolds, nano-HA particle-incorporated scaffolds implanted to rat femoral defects promote the M2 phenotype and enhance bone regeneration and vascularization [194]. Liang et al. [196] also found that mesoporous silica loaded with gold nanoparticles can promote polarization transition and stimulate macrophages to secrete osteogenic cytokines for osteogenesis.

#### 4.1.5. Biophysical Stimuli

In addition to the above biochemical stimuli, which can regulate immune cells to promote bone regeneration, biophysical stimuli have immunomodulatory functions and can be categorized into internal structural stimuli, external mechanical stimuli, and electromagnetic stimuli [7].

Internal structural stimuli refer to cues derived from the unique biophysical properties of biomaterial scaffolds. A myriad of internal structural stimuli has been revealed to show osteoimmunomodulatory effects, which include matrix stiffness, pore size and porosity, surface hydrophilicity, surface roughness and topography, and surface charge [170]. The modulation of immune cells by internal biophysical stimuli to promote bone regeneration shows potential for critical bone defects. Ni et al. [197] designed and compared two patterns of nanotopography for osteogenesis and angiogenesis: nanoconcave pit (NCPit) and nanoconvex dot (NCDot) microarrays. They revealed that NCDot microarrays dramatically promote M2 macrophage polarization with higher anti-inflammatory markers (TNF-*α*, IL-6, IL-1*β*, and CD 86) and lower inflammatory cytokines (IL-10 and CD 206) than NCPit microarrays [197]. Therefore, NCDot microarrays can considerably promote the osteogenic differentiation of MSCs because of an appropriate immune microenvironment [197]. Another group developed hierarchically structured (microchanneled) three-dimensional (3D) scaffolds [198]. In comparison with traditional 3D-printed scaffolds, microchanneled scaffolds can inhibit the extracellular trap formation of anchored neutrophils, promote M2 macrophage polarization, and improve the expression of SDF1 to recruit MSCs and VEGF to enhance vascularization [198]. Then, in vivo experiments have shown that scaffolds can dramatically enhance bone regeneration and reduce fibrous capsule formation [198]. Therefore, the application of internal structural stimuli may be one ideal approach to replace biochemical stimuli because they may avoid burst release and provide microenvironments for bone regeneration.

External mechanical stimuli and electromagnetic stimuli are cues executed by outside biophysical effects but not the structural properties of biomaterials. Dong et al. [199] found that mechanical tension can promote the polarization of M2 macrophages, which secrete anti-inflammatory factors, including IL-10 and TGF-*β*, to promote the osteogenic differentiation of MSCs. Low-intensity pulsed ultrasound can also stimulate the switch of an inflammatory M1 phenotype to an anti-inflammatory M2 phenotype [200]. Considering related studies on external mechanical stimuli and electromagnetic stimuli to regulate immune cells are limited, further studies should focus on the immunomodulatory functions of other external mechanical stimuli (such as compression and fluid flow shear stress) and electromagnetic stimuli (such as electric current, electric field, magnetic field, and electromagnetic field) for bone regeneration.

### 4.2. Modulations of MSCs to Affect Immune Cells for Bone Tissue Engineering

Considering that MSCs are generally used as repair cells for bone tissue engineering and used as immunomodulators because of their unique properties of low immunogenicity, immune stimulation, and immunosuppressive effects [170], MSC modulation is another strategy to affect immune cells for bone tissue engineering.

In general, MSCs are seeded on scaffolds, which are then transplanted to bone defects for bone regeneration. Seebach et al. [129] found that cultured MSCs promote the recruitment of M1 macrophages and endothelial progenitor cells to scaffolds, which allow for initial maturation and early vascularization. Therefore, the transition of the M1 phenotype to the M2 phenotype is important for MSC-seeded scaffolds, and modulating MSCs to secrete bioactive factors may be a potential approach. Ueno et al. [201] fabricated lentivirus-transduced IL-4-overexpressing MSCs, which are loaded to scaffolds for critical bone defects. They demonstrated that modified MSCs embedded in scaffolds can promote M2 macrophage polarization without influencing the M1 macrophage activity at the early inflammation phase [201]. IL-4-generated scaffolds can promote bone regeneration, revealing that scaffolds loaded with modified MSCs may be a promising strategy [201]. However, the M1 activity of MSC-seeded scaffolds is high because of the matrix produced by MSCs [202]. Thus, the choice of MSCs may be future priorities.

In addition to the direct loading of MSCs to scaffolds to regulate immune cells, MSCs can be systemically infused to diminish inflammation. Liu et al. [203] found that inflammatory cytokines (IFN-*γ* and TNF-*α*) at implantation sites are downregulated by the systemic infusion of MSCs because this method can upregulate Tregs. This approach can also promote bone regeneration in MSC-seeded scaffold [203]. A systemic review has shown that the systemic infusion of MSCs can promote bone regeneration in animal models [204]. However, detailed mechanisms should be explored in future studies.

## 5. Conclusions

This review mainly focuses on the interaction between MSCs and immune cells and related modulations for bone tissue engineering. Under specific conditions, immune cells can promote the recruitment, proliferation, and osteogenic differentiation of MSCs. MSCs can also exert immunomodulatory effects on various cells. In targeting the interaction between MSCs and immune cells, different modulations administer immune cells or MSCs to promote bone regeneration for critical bone defects. Biochemical stimuli (bioactive proteins or peptides, nonamino acid drugs, metal ions, and nanoparticles) and biophysical stimuli (internal structural stimuli, external mechanical stimuli, and electromagnetic stimuli) can be used to regulate immune cells to promote MSC osteogenesis. Modified MSCs and systemic MSC infusion can be utilized to regulate immune cells for bone regeneration.

Although some advances have been made in the interaction between MSCs and immune cells, a more detailed cross-link is necessary to be interpreted because it may provide a beneficial basis for surgical bone repair by bone tissue engineering. Currently, the modulations of immune cells to affect MSCs for bone regeneration are mainly based on stimulating the switch from M1 macrophage polarization to M2 macrophage polarization. However, the appropriate M1 macrophage activation of the initial phage may also benefit bone regeneration. Therefore, future studies should focus on the sequential polarization of M1 and M2 macrophages. Immune cells other than macrophages participate in the interaction between MSCs and immune cells, so modulations toward other immune cells to affect MSCs for bone regeneration should be also developed. Furthermore, modified MSCs show potential for application in bone tissue engineering, but biosafety issues should be resolved. In brief, surgical bone repair by bone tissue engineering should consider the interaction between MSCs and immune cells. Thus, this technique may ultimately promote the clinical treatment of bone tissue engineering.
